# Alien Roadside Species More Easily Invade Alpine than Lowland Plant Communities in a Subarctic Mountain Ecosystem

**DOI:** 10.1371/journal.pone.0089664

**Published:** 2014-02-26

**Authors:** Jonas J. Lembrechts, Ann Milbau, Ivan Nijs

**Affiliations:** 1 Research Group of Plant and Vegetation Ecology, Department of Biology, University of Antwerp, Wilrijk, Belgium; 2 Climate Impacts Research Centre, Department of Ecology and Environmental Science, Umeå University, Abisko, Sweden; University of Tartu, Estonia

## Abstract

Effects of roads on plant communities are not well known in cold-climate mountain ecosystems, where road building and development are expected to increase in future decades. Knowledge of the sensitivity of mountain plant communities to disturbance by roads is however important for future conservation purposes. We investigate the effects of roads on species richness and composition, including the plant strategies that are most affected, along three elevational gradients in a subarctic mountain ecosystem. We also examine whether mountain roads promote the introduction and invasion of alien plant species from the lowlands to the alpine zone. Observations of plant community composition were made together with abiotic, biotic and anthropogenic factors in 60 T-shaped transects. Alpine plant communities reacted differently to road disturbances than their lowland counterparts. On high elevations, the roadside species composition was more similar to that of the local natural communities. Less competitive and ruderal species were present at high compared with lower elevation roadsides. While the effects of roads thus seem to be mitigated in the alpine environment for plant species in general, mountain plant communities are more invasible than lowland communities. More precisely, relatively more alien species present in the roadside were found to invade into the surrounding natural community at high compared to low elevations. We conclude that effects of roads and introduction of alien species in lowlands cannot simply be extrapolated to the alpine and subarctic environment.

## Introduction

Roads have major effects on the ecosystems they cross [1]–[5]. They alter species composition in roadsides through habitat fragmentation [1], [5], enhanced propagule dispersal (transportation of plant seeds by cars, animals and footwear [6]–[9]) and through changes in biogeochemistry (soil pH, nutrient status), hydrology and erosion [1], [2], [10], [11]. These processes withhold local species and promote the establishment of disturbance-tolerant, ruderal, and competitive species [1], [12]–[15].

Roadside edges are mostly characterized by greater plant species richness than their surroundings [16]–[18], although the promotion of these plant strategies and the local extinction of species poorly adapted to roadsides homogenize the roadside communities [13]. Also alien species are known to be good colonizers of roadsides, and alien species pools accumulating in roadsides may be a source for subsequent invasion into the surrounding natural community [14], [19], [20].

The influence of roads gradually declines with increasing distance to the road [16], ending at several to hundreds of meters or more. Size, usage and building material of the road all determine the depth of the edge effect [16], [18], [21], [22]. Together with the vegetation type, these factors also influence the invasibility of the surrounding natural communities [14], [19], [23]. As an example, deeper edges are for instance found in boreal compared to temperate woods [16], [24].

While road-based effects on plant communities are well studied in general, less is known of the effect of roads on (sub)arctic mountain ecosystems, where typical roadside species might be limited by low temperatures and natural communities change rapidly over short elevation distances.

Research on roadside plant communities in mountains has mostly focused on trends in species richness, thereby often comparing patterns of native and alien species. With increasing elevation, native species richness in roadsides follows a hump-shaped [13], [25] or decreasing [17] pattern. In alien plant species richness along mountain roads, a strong decline of species richness is found [26]–[28] (but see [17]). The specific cause of this decline in alien species with increasing elevation is subject of debate. Either it is due to increasingly harsh abiotic conditions [27], a low propagule pressure in the less anthropogenic highlands [26], [29], or simply the lack of time to colonize higher elevations since recent introductions in the lowlands [30], [31]. The resistance of the resident plant communities [32] and how this changes with increasing elevation is also expected to play a role, but has been little studied in relation to the spread of alien species from mountain roads. Although with increasing elevation less alien species are found in natural vegetation [33], it is unclear whether this results from a smaller number of aliens in high elevation roadsides, or from a lower invasibility of high-elevation natural communities. How roads influence community composition in mountains in general, and whether and how the effect of roads changes with increasing elevation is still unexplored.

This is the first study of the effects of roads on the composition of plant communities in subarctic mountains, where climate conditions provide limitations from low elevations onwards, and where there are short and steep gradients to the alpine zone. Our objectives were to determine (1) changes in the effects of roads on species richness and composition along elevational gradients, including the plant strategies that are most affected, (2) whether and how mountain roads promote the introduction and invasion of alien plant species in subarctic ecosystems.

## Methods

### Study sites

The study was carried out in July 2012 in the Northern Scandes, in the vicinity of Narvik, Norway, 220 km north of the Arctic Circle (68°26’18” N, 17°25’40” E). We selected three comparable mountain roads going from sea level to ca. 720 m a.s.l. (the tree line in the area is situated at ca. 600 m a.s.l.). The roads were constructed in the eighties, and host both tourist traffic and regular summer traffic of trucks to hydropower plants in the mountains. The roads were gravel covered, in good condition and flanked with a drainage system; on one road, gravel addition was ongoing during the sampling period. No specific permissions were required for these locations and activities.

The field studies did not involve endangered or protected species.

The Norwegian west coast profits from the relatively warm North Atlantic Current, giving the lowlands a subarctic oceanic climate with an average annual temperature and precipitation of 3.8°C and 830 mm, respectively, and average July and January temperatures of 13°C and –4°C [34]. The lowland vegetation is characterized by forests dominated by mountain birch (*Betula pubescens* Ehrh. ssp. *czerapanovii*), willow (*Salix* sp.), and pine (*Pinus sylvestris* L.) with an understory of mainly ferns (e.g. *Dryopteris expansa* (C. presl) Fraser-Jenk & Jermy and *Gymnocarpium dryopteris* (L.) Newm.). On the mountain slopes, climatic conditions are drier and colder and from *ca.* 150 m a.s.l. the vegetation changes to nutrient-poor open forests with a dense understory dominated by bryophytes, *Empetrum nigrum* ssp. *hermaphroditum* L. and *Vaccinium* species (*V. uliginosum* L., *V. vitis-idaea* L. and *V. myrtillus* L.). Above the tree line, at around 600 m a.s.l., we found alpine vegetation dominated by bryophytes, dwarf shrubs (*Betula nana* L., *Vaccinium* sp., *E. nigrum*) and graminoids. The climatic and vegetation gradient thus shifted from productive, boreal lowland forests to poor alpine vegetation over a span of 720 m.

### Data collection

**Figure 1:**
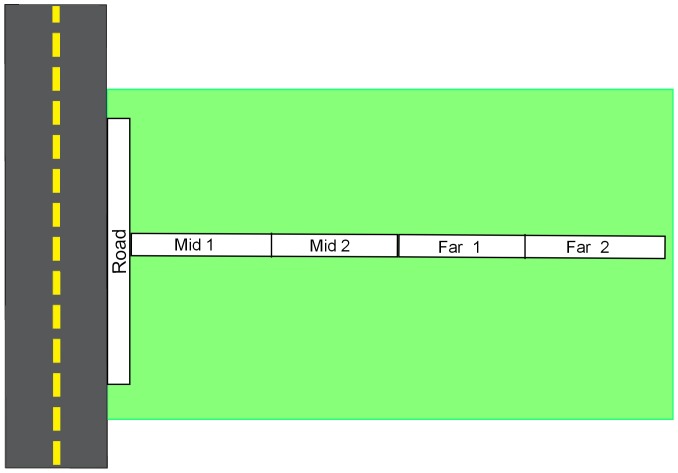
Transect outline. Transects were replicated 20 times along each of the 3 roads. Each plot (road, mid, far) measured 2×50 m, with the roadside plot parallel to the road, and the mid and far plots perpendicular. Mid and far plots both are divided in two subplots of 2×25 m each (mid1, mid2, far1 and far2).

Data collection followed the design of the Mountain Invasion Research Network (MIREN; www.miren.ethz.ch [35]). Along each of the three roads, 20 T-shaped transects were selected with an altitudinal interval of 30 to 35 m (Fig. 1). Every transect consisted of three adjoining plots, 2×50 m each, one parallel (adjacent) to the road (‘road’), and two perpendicular to it (‘mid’, ‘far’). The far plots, ranging from 52 to 102 m away from the road, were considered to contain the natural plant communities. The plots perpendicular to the road were subdivided into subplots of 2×25 m (mid1, mid2, far1, far2; Fig. 1). The parallel roadside plot was placed at the first occurrence of roadside vegetation. Side of the road was decided at random, if not prevented by relief, impassable rivers or hairpin bends. Elevation and geolocation of transects and plots were recorded with a GPS in the middle and at the end of each plot.

We recorded the cover of all native and alien vascular plant species at the plot level, and additionally species occurrence in subplots. Species were identified with Den nya nordiska floran [36], and species status (native or alien) was determined with the help of national and international databases and expert advice [37]–[42]. We define aliens as introduced from another region into the northern third of Norway after 1492. We used this cut-off date to allow comparison with the other research within the MIREN-consortium [27], [33]. Due to dissimilarities in the definition of aliens in the different sources, a species was only considered an alien if stated as such by at least 2 independent sources on a national [37]–[40] or regional [41], [42] scale (see Appendix S1).

In each plot, we noted mountain zone (lowland, montane, alpine) and habitat (forest, open forest, shrubland, forb-grassland, riparian, rock). Cover classes (0–1%, 1–5%, 5–25%, 25–50%, 50–75%, 75–95%, 95–99%, 99–100%) were used to estimate by eye the amount of bare ground, total vegetation, disturbance, moss cover, creeks/rivers (open water), moisture (marshes and wetlands) and forest canopy. The centers of these cover classes were used in the statistical analysis.

Grime’s triangle [43] was used to classify species following their ecological strategy (ruderal, competitive or stress-tolerant). Specific species values were derived from floraweb.de [44]. When a native species showed traits of different strategies, the percentage for each category was estimated [45], [46] and used to weigh native species richness of a plot in the analysis. For example, species with a strategy of CSR, in the middle of the triangle, would have a score of 0.33 for each strategy.

All data will be made available through the global MIREN Invasive Species Database (www.miren.ethz.ch [35]).

### Data analysis

The relation between species richness and the aforementioned explanatory variables (mountain zone, elevation, distance to road, habitat, the amount of bare ground and disturbance and the cover of total vegetation, bryophytes, creeks/rivers, moisture and forest canopy) was analyzed with (Generalized) Linear Mixed Models (GLMMs, R-function: glmer/lmer[47]). GLMMs assuming a Gaussian distribution were used for native species richness, while alien species richness was best approximated with a Poisson distribution. We more specifically tested elevation, distance to the road and their interactive effect on native species richness and alien species cover (Gaussian distribution) and on ruderal, competitive, stress-tolerant and total alien species richness (Poisson distribution).

Transect and road identity were tested as random factors, with transect nested in road identity. In the analyses of native species richness and alien species cover, the log likelihood ratio showed significance for transect (p<0.05), so transect was kept in as a random effect. The models of alien species richness, cover and the alien species strategies did not show a significant log likelihood ratio and could be simplified to a regular generalized linear model without random effects (glm). The Akaike Information Criterion (AIC) was used to identify the GLMs with the best fit, comparing anova’s was used for the GLMMs.

Cover percentage of total vegetation, mosses, *E. nigrum* and bare ground were tested with a glm (Gaussian distribution) as a function of distance to the road, elevation and their interaction. To analyze native and alien species richness in the subplots, we made use of generalized linear models (glm) with distance to the road as a factor and compared the results with a Tukey post-hoc test (TukeyHSD).

Total species composition was analyzed with a Detrended Correspondence Analysis (DCA), based on the cover (%) of each species in each plot (decorana in the VEGAN package [48]). Plots were grouped into 9 assemblages according to all combinations of elevation (low, mid, high) and distance to the road (road, mid, far). Ellipses of standard deviation were calculated to indicate significance (ordiellipse in the VEGAN package).

To understand how roads eliminate local species and allow for new ones, we counted the number of both native and alien species that were present in the natural plant communities (far plots) but missing in the roadside, the number of species that were newly gained in the roadside, and the number of shared species between the roadside and the natural communities (per transect). These species counts were analyzed in linear models as a function of elevation, separately for ruderal, competitive and stress-tolerant species.

Relative alien species richness in the natural plant communities was calculated as the ratio of alien species richness in the more distant plots (‘mid’ and ‘far’) to alien species richness in the roadside plot of the same transect. This parameter indicates the extent to which the roadside alien species pool represents a source for invasion into the neighboring natural plant communities. Relative alien species richness was examined with a linear model with elevation as explanatory variable.

Finally, we investigated whether maximum elevation (highest record) and elevational range (highest minus lowest record) were related across native and alien species. This relationship indicates the degree to which species can occupy the elevational gradient, taking into account the environmental barriers that constrain occurrence at higher elevation. Differences in range between natives and aliens were compared with a linear model containing both highest occurrence, status (native or alien) and their interaction. All statistical analyses were performed in R [49]. Differences were considered significant if p<0.05.

## Results

### Native species

A total of 210 species were identified, of which 196 were native to the region. The best fitting GLMM-model for native species richness contained habitat factors (creeks/rivers, moisture, habitat type, percentage of vegetation cover), elevation, distance to the road and bryophyte cover (AIC = 1196.1, AIC = 1203.1 for the model containing all variables). Bare ground, disturbance and forest canopy did not explain any additional variation.

**Figure 2:**
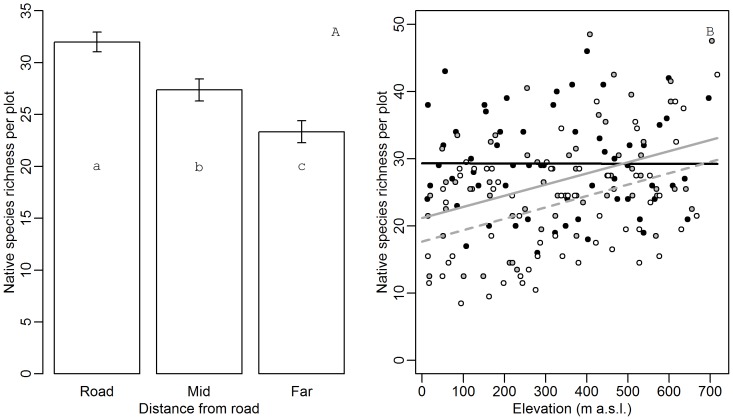
Native species richness as a function of elevation and distance to the road. (A) Average native species richness (±1 SE) in plots across the elevational gradient. Different letters indicate significant differences (p<0.05) in a Tukey’s post-hoc test. (B) Native species richness (number of species per plot) as a function of elevation. •, black full line: roadside plots; •, grey full line: mid plots; ○, broken line: far plots (see Fig. 1 for plot types).

Averaged over the three elevational gradients, roadside plots had higher native species richness than plots far from the road (p<0.001), while in-between plots had intermediate richness (Fig. 2a). This intermediate richness originated from higher richness in mid1 (the subplot closest to the road), while species richness in mid2, far1 and far2 did not differ (p = 0.001, 0.02, <0.001 for mid1 compared with mid2, far1 and far2, respectively; p>0.05 for mid2, far1 and far2 mutually). However, these differences between roadside and natural vegetation disappeared with increasing elevation due to an interaction between elevation and distance to the road (Fig. 2b, p = 0.02 for both mid and far), as species richness increased in mid and far plots only and remained constant in roadside plots.

**Figure 3:**
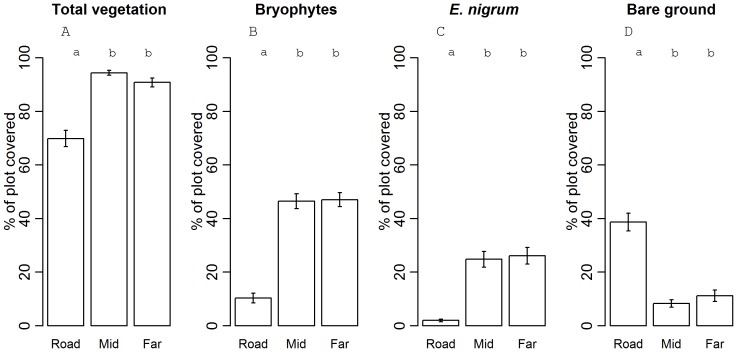
Plot coverage as a function of distance to the road. Percentage of the plots (± 1 SE) covered with (A) total vegetation, (B) bryophytes, (C) the dominant species (*E. nigrum*) and (D) bare ground, as a function of distance to the road. Different letters indicate significant differences (p<0.001).

To understand these differences in species richness with distance to the road, we tested variables correlated with roadsides (Fig. 3) and elevation. Because no interaction between distance and elevation was found, the interaction term was left out. Roadside vegetation cover was 21–25% lower than in intermediate and far plots (Fig. 3a, p<0.001), which was reflected in a drop in percentage cover of bryophytes and *E. nigrum* (the most common dwarf shrub of the natural plant communities) to close to zero (Fig. 3b and 3c, p<0.001). The amount of bare ground, on the other hand, increased with 30% in the roadsides (Fig. 3d, p<0.001). For none of these variables, significant differences between intermediate and far plots were observed (p>0.05). Only for bare ground a significant increase with elevation was found (p<0.001). Vegetation cover declined simultaneously (p = 0.03).

**Figure 4:**
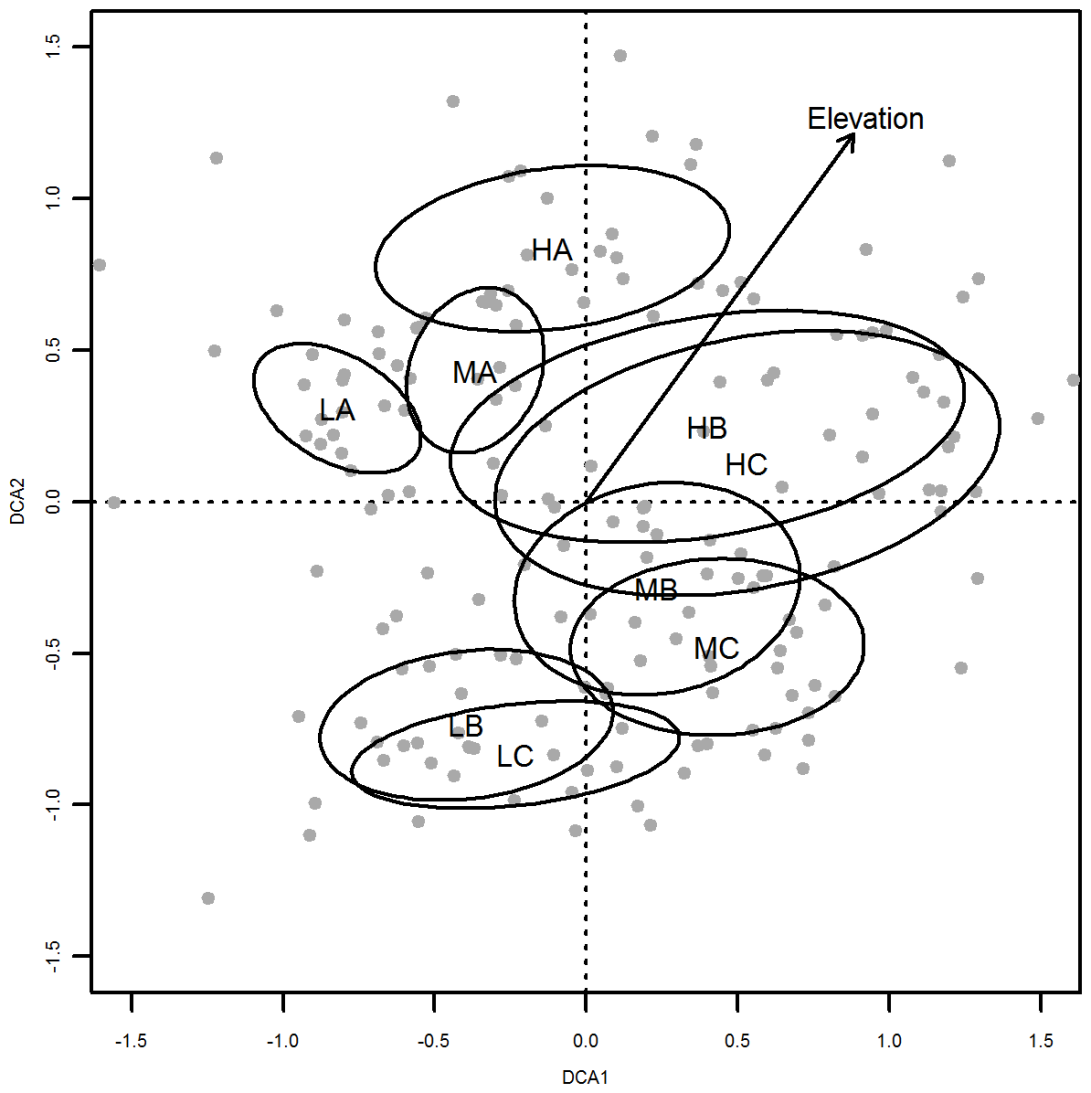
Ordination of plots. DCA-ordination of plots (•) based on total species coverage. Ellipses indicate the standard deviations for different subgroups as a function of elevation and distance to the road. Elevation: H = highest third, M = middle third, L = lowest third of the gradient; road distance: A = roadside, B = mid, C = far (see Fig. 1 for plot types). The arrow represents the vector of increasing elevation. Eigenvalues of DCA1 and 2 are 0.3479 and 0.2771 respectively.

The interacting effects of elevation and distance to the road on species richness in Fig. 2b were explored further by analyzing species composition (Fig. 4). The DCA clearly distinguished between roadside communities (A) and communities close to (B) and far away (C) from the road (Fig. 4). Intermediate plots (B) had a species composition shifted slightly towards the roadside communities. The species composition changed gradually with increasing elevation, as shown by the arrow. On high elevations (H), species composition in both intermediate (B) and far (C) plots differed less from the roadside community than in the lowlands, confirming the pattern found in species richness. However, the larger ellipses indicate on average more variation and hence a larger heterogeneity on high elevations.

**Figure 5:**
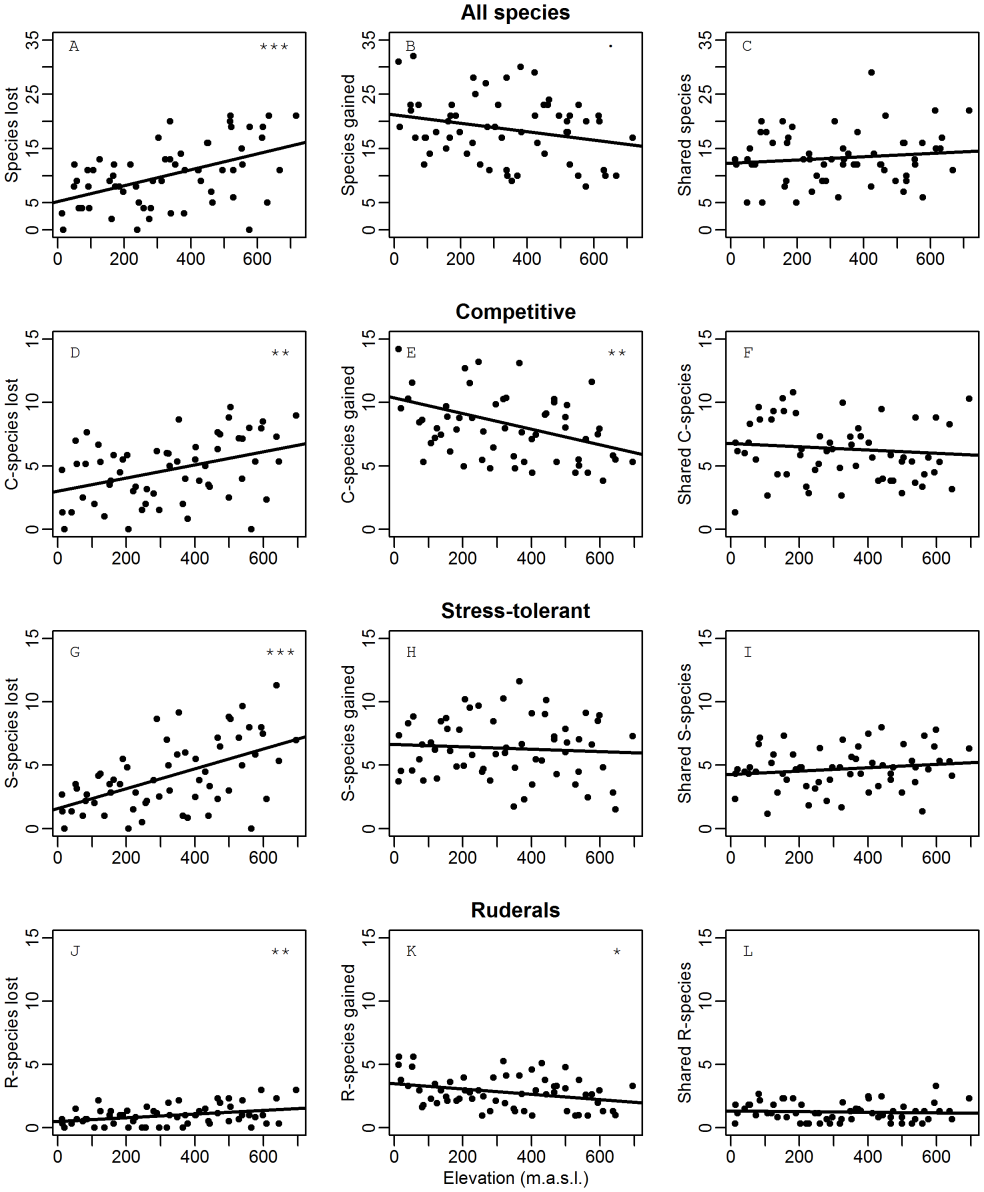
Changes in total species composition between roadside and far plots with elevation. Left column: number of species that were present in the natural plant communities but lost in the roadsides ( =  species unique for the natural plant communities). Middle column: species newly gained in the roadsides ( =  species unique for the roadsides). Right column: shared species between roadsides and natural plant communities. A-C  =  all species, D-F  =  competitive species, G-I  =  stress-tolerant species, J-L  =  ruderals, derived from Grime’s triangle. Significance of linear regressions: ***: p<0.001; **: p<0.01; *: p<0.05;.: p<0.1; otherwise higher than 0.1.

To get a more detailed view of these shifts in community composition, we investigated the extent to which local species were eliminated in the roadside and, conversely, the extent to which the roadside was enriched with new species which did not occur in the natural plant communities (Fig. 5). On high elevations, more species from the natural plant communities were absent from the roadsides (‘species lost’, Fig. 5a, p<0.001). However, the number of species unique for the roadsides (‘species gained’, Fig. 5b) decreased higher in the mountains (p<0.05). The number of shared species, occurring both in roadsides and in the natural plant communities, was insensitive to elevation (Fig. 5c, p>0.05). All three plant strategies from Grime’s triangle showed greater losses from the natural plant communities on high elevations, though the increase was most obvious for the stress-tolerant species (Fig. 5d,g,j, p<0.001 for S, p between 0.001 and 0.01 for C and R). Conversely, the greater roadside species gain in lowlands relative to highlands was caused by more new competitive and ruderal species (Fig. 5 e,k, p between 0.01 and 0.05), but not by a higher gain of stress-tolerant species (Fig. 5h, p>0.05). On high elevations, species gained in roadsides were mostly stress-tolerant species, while competitive and ruderal species were less abundant. In shared species, no significant trends with respect to plant strategies were observed (Fig. 5 f,i,l, p>0.05).

### Alien species

Based on national databases, 11 aliens were identified (Aegopodium podagraria L., Festuca pratensis Huds., Phleum pratense L. ssp. pratense, Plantago major L., Poa annua L., Poa pratensis L. ssp. pratensis, Stellaria graminea L., Trifolium repens L., Trifolium pratense L., Tanacetum vulgare L., Vicia cracca L.). Three other species were added as regional aliens for northern Norway: Achillea millefolium L., Agrostis capillaris L. and Picea abies (L.) H. Karst). Two species were defined as aliens but left out of the analyses due to ambiguities with the determination of subspecies (Anthoxanthum odoratum L. and Taraxacum officinale L.). All aliens are from European or Eurasian origin [40]. For details on aliens, consult Appendix S1.

64% of the aliens followed the competitive strategy, none of them were stress-tolerant species, only 7% were ruderals and the remaining 29% were generalist species (CSR-strategy). Because of the important fraction of generalists, they were used as an extra category in the analysis, without using the weighing factor. In general, differences in alien species richness and cover were best explained by elevation, distance from the road and native species richness (AIC  =  355.44 for the GLMM of species richness, vs. 379.29 for the model containing all variables, AIC  =  938.54 vs. 956.16 for species cover). Adding the interaction between elevation and distance from the road lowered the AIC further to 354.05 and 936.05 respectively. The interaction is a result of a strong decrease in aliens with elevation in roadsides, but a simultaneous increase in the natural vegetation (p<0.001 and p = 0.03 for alien species richness in mid and far plots, respectively, p = 0.03 and p = 0.03 for alien cover in mid and far plots).

**Figure 6:**
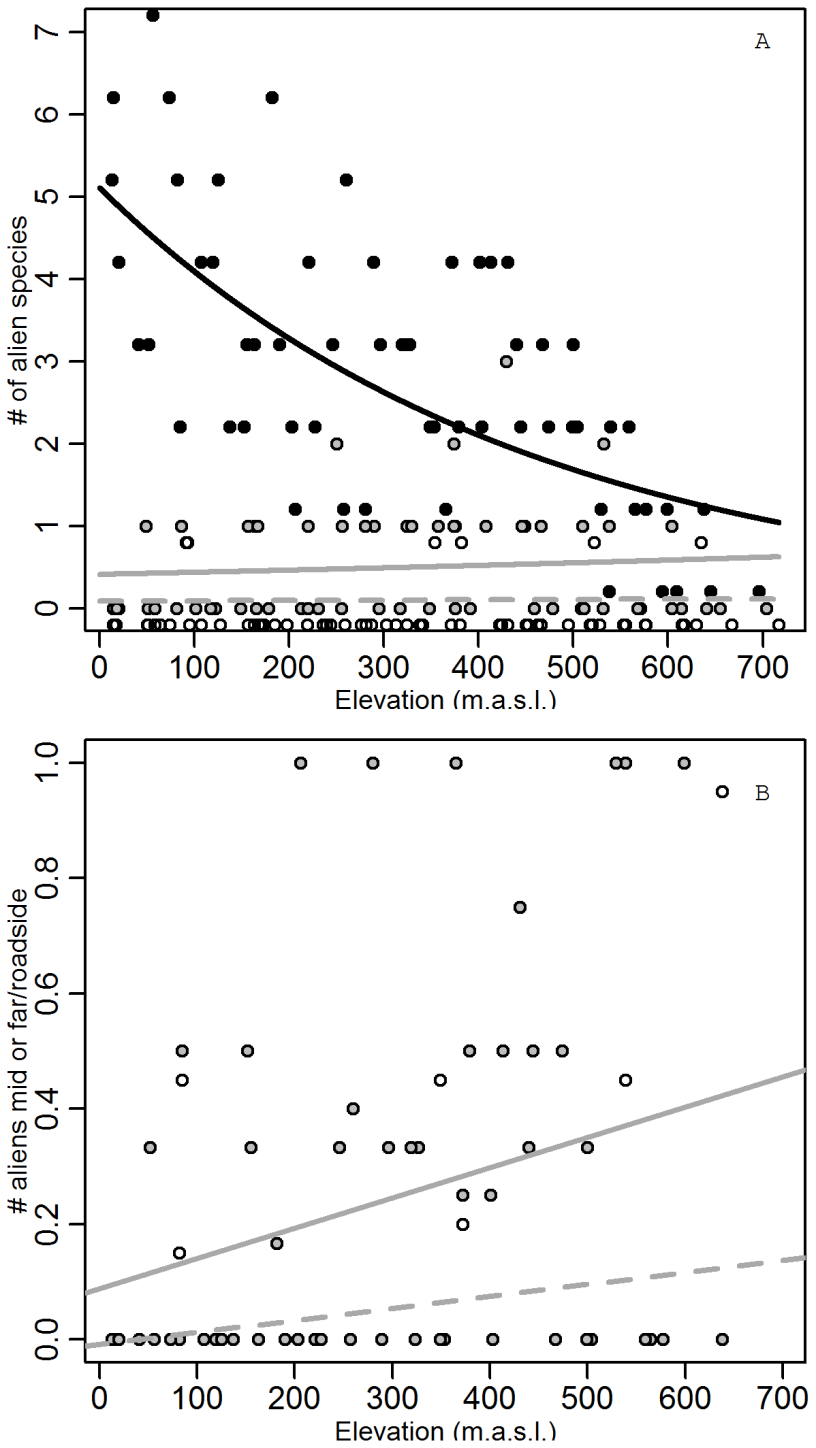
Alien species richness as a function of elevation and distance to the road. (A) Alien species richness (number of species per plot) as a function of elevation. Roadside plots (•, black line), intermediate plots (•, grey line) and far plots (○, dashed grey line). (B) Ratio of alien species richness in the natural plant communities to that in the roadside plot, with mid/roadside (•, grey line) and far/roadside (○, dashed grey line). Significance of linear regressions: see text. Symbols of different variables were slightly shifted to avoid overlap.

These contrasting trends between aliens in roadsides and the natural plant communities resulted in an increased relative alien species richness in the mid and far plots towards higher elevations (Fig. 6b, p = 0.01). On higher elevations, a larger fraction of roadside aliens could thus successfully invade the natural plant communities. This increase in invasion was larger in intermediate than in far plots.

CSR-types of the aliens changed over the elevational gradient, with competitive aliens being more abundant in the lowlands and decreasing with elevation (p<0.001). On high elevations, they were replaced by an increasing relative amount of generalists (CSR type, p = 0.003). No interactions with distance to the road were found.

**Figure 7:**
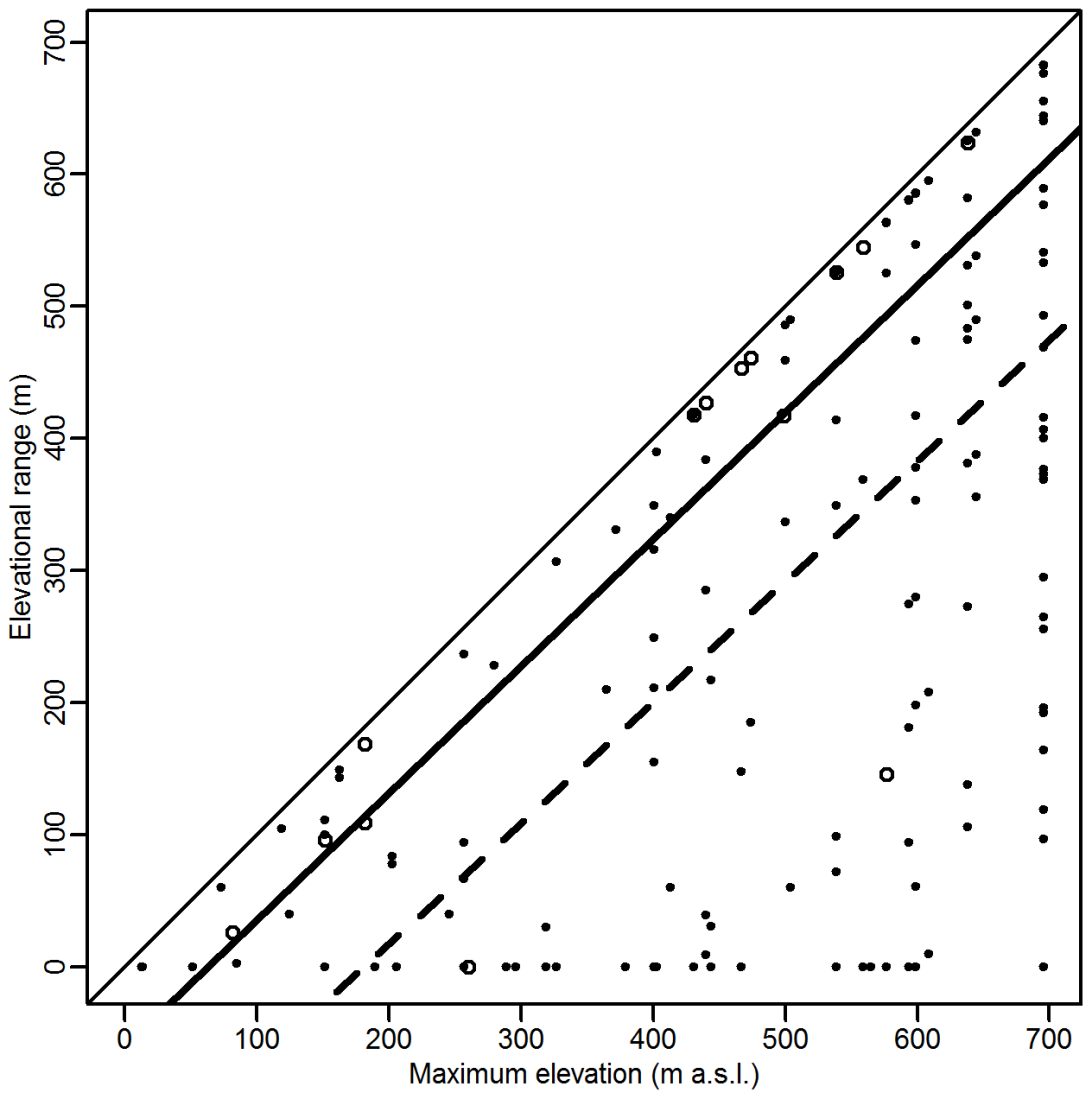
Elevational range of native and alien species. Relationship between elevational range and maximum elevation reached by native (•, dashed line) and alien (○, thick solid line) species. Symbols are constrained to the lower right of the graph (marked by the thin solid line), since the elevational range cannot exceed the maximum elevation.

Alien species had a wider elevational range than native species, when species with the same maximum elevation were compared (Fig. 7, p = 0.02). This implies that the range of the aliens started at a lower elevation, hence these species were largely only constrained by the conditions higher in the mountains. In native species, the range was randomly distributed between small and wide and distributional constraints were present also at lower elevations. The interaction between elevation and status was not significant.

## Discussion

### Native species

Roadside edges in the subarctic mountains contained more species than the natural plant communities, which is similar to other ecosystems [14]–[18] (but see [12]). Beside a potentially higher propagule pressure and better abiotic growing conditions (higher temperature, more light and nutrients and altered hydrology) in roadsides [2], [10], [14], [50], our results point to a succession setback as probable cause for this greater richness. This conclusion is supported by the observed reduced vegetation cover and increased amount of bare ground in the roadside communities, which could provide more opportunities for germination. Especially the cover of *E. nigrum* ssp. *hermaphroditum*, a highly dominant clonal shrub known to impair the establishment of other species owing to its dense structure and production of allellopathic compounds [36], [51], [52] was significantly lower in the roadsides (2%) compared to the natural plant communities (26%). Also a similar decrease in (dense) moss cover in the roadside (from 47% to 10%) can be linked to higher germination and establishment chances [53], [54]. We therefore put forward that, even in harsh subarctic ecosystems, changes in community structure and plant-plant interactions may contribute to the impact of roads, in addition to dispersal-related and abiotic changes.

Unexpectedly, the divergence in species richness between roadside plots and the natural community diminished when elevation increased. This pattern originated from enhanced species richness in the natural plant communities, while the roadside species richness remained approximately constant. The enhanced species richness at higher elevation in the natural communities might be attributed to the higher availability of bare ground for germination and the greater habitat diversity. Nevertheless, these greater species pools in the natural plant communities did not entail greater richness in adjacent disturbed roadside communities.

The species composition in the roadside changed along the gradient, and did thus not consist of a fixed set of typical roadside species. Notably the input of competitive and ruderal species that occurred exclusively in the roadside plots (i.e. which were not found in the natural plant communities) weakened higher in the mountains. The premise that mostly competitive and ruderal species benefit from roads [12], [14], [15] would thus not necessarily hold in alpine environments (and perhaps neither in other stressful environments). Instead, the fraction of stress-tolerant species became more important, although this co-occurred with a higher loss of stress-tolerant species from the natural plant communities. This suggests that roadsides on high elevations serve as a refuge for a variety of alpine stress-tolerant species (although not necessarily the same species as in the nearby natural vegetation) rather than containing a large pool of ruderal and competitive species, some of which might be prone to invasion (see below).

The edge effect on species richness had a limited spatial extent, only affecting the first subplot of the natural plant communities (Fig. 1) up to 27 m from the road. This matches the extensive use of the road [22], which limits the physical disturbance of bryophytes and dwarf shrubs needed for the germination of other species.

### Alien species

The observed association of aliens with lowland roadsides [17], [28], [55] and the general decrease in alien species richness and coverage with elevation [13], [26], [27], [33], [55]–[59] are consistent with other studies, although the hump-shaped pattern of invasion is missing in our data. A possible explanation is the absence of limiting growing conditions in the lowlands that often create such a pattern in other study regions [13]. In the studied subarctic mountain range, the best growing conditions occurred on the lowest elevations.

The decline of alien species richness and coverage in the roadsides with increasing elevation and the wider elevational range of alien species compared to natives hint to patterns of invasion similar to those in literature [27], [58]: mountains act as environmental filters, with aliens establishing first in the lowlands. Only species that are successful under lowland climatic conditions can subsequently invade the mountains [30]. Environmental constraints [27], [33], [59], a lower propagule pressure [32] or decreased human land use (hence lower disturbance [33]), provide a progressive drop-out of alien species with increasing elevation. This limits their occurrence higher in the mountains. We found this theory to hold true especially for competitive species, while generalist species managed to reach higher elevations in the mountains. Seipel et al. [33] showed that these factors in some cases also result in a greater absolute loss of alien species away from the road on higher elevations.

While our findings are largely in agreement with those of previous studies, a different perspective is provided by the observed interaction between elevation and distance to the road, as well as by our calculation of relative alien species richness (natural communities vs. roadsides). Relative alien species richness separates the influence of the roadside alien species pool (which depends on the lowland alien species pool and the ecological filtering by the mountain) from the influence of the invasibility of the alpine system. We observed greater absolute and relative alien species richness and cover in the alpine system than in lowlands, suggesting higher invasibility (in contrast to [33]). Invasive escape was greater on higher elevations, even though the alien roadside species pool – and thus propagule pressure - on these elevations was smaller due to ecological filtering [27], [60]. Because plant strategies of alien species were unrelated to distance to the road and no interaction with distance and elevation was found for plant strategies, we expect this higher invasive escape on high elevations to be independent from alien plant traits (and thus invasiveness), but to originate rather from a higher invasibility of the alpine habitat. A higher invasibility can result from (1) more variable vegetation, (2) higher native species richness, (3) a lower vegetation cover or (4) an evolutionary history of low competition.

A replacement of the common heathland vegetation by a more heterogeneous vegetation type could enhance invasibility, as heathland is the least invasible vegetation type in the subarctic [61]. Although we did not find a lower cover of *E. nigrum* or mosses on high elevations, the alpine vegetation showed more variation in species composition. This more variable landscape can create more opportunities for invasion than the less heterogeneous lowlands.

With this higher variation comes higher native species richness. In agreement with other observational studies on this spatial scale [62]–[64] (but see [65], [66]), this higher native species richness is linked to increased invasion. Possible explanations are a more heterogeneous environment [63], or non-equilibrium conditions [62], [67]. Because of the previously shown correlation between alien and native diversity and a lower presence of the typical subarctic climax vegetation in our study plots, these explanations have a high probability. While the heterogeneous environment will be the main driver of the invasion in the natural vegetation on high elevations, the invasion in roadsides is more likely linked to non-equilibrium conditions.

A third possible explanation is the creation of empty niches through the higher availability of bare ground [62], [68], [69]. While we consider this an important driver of invasion in the roadsides on low elevations, it is less likely to play an important role in the alpine environment. Conditions on bare ground in the climatically harsh alpine environment are less suitable for invasion, which may rather acquire facilitation [70]–[74].

Because of these higher importance of facilitation compared to competition in alpine conditions, the evolutionary history has shown relaxed selection for competitive ability [64]. This would imply that the alpine habitat is evolutionary not prepared to deal with the increased disturbance caused by the building of the roads and the introduction of species with more competitive abilities.

The conclusion that alpine habitats are more vulnerable to invasion adds to the growing evidence that risk of invasions in mountains may increase in a future with greater alien propagule pressure, as previous research showed that alien species germination is not likely to be affected by harsh climate conditions in mountains [61] and aliens are still expanding their range in the mountains [31].

## Conclusion

The structure of mountain plant communities, the introduction of aliens in native communities, and roadside edge effects on plants have all been extensively studied in ecology. The integration of these community properties and processes, as well as the location of the current study in the subarctic environment, provides new input for the debate on their possible interaction. Our results suggest that alpine plant communities react differently to road disturbances than their lowland counterparts. The roadside plant communities on high elevations differed less from the local natural community and contained less competitive and ruderal species compared with lower elevations. However, invasion by aliens into the natural vegetation occurred relatively more at high elevations, even though the alien species richness in mountain roadsides was lower. This higher invasibility on high elevations can be linked to a higher diversity in abiotic and biotic conditions and a relaxed selection for competitive ability in the alpine system. This highlights the fact that effects of roads on alien introduction in lowlands cannot simply be extrapolated to the alpine and subarctic environment.

## Supporting Information

Appendix S1 **Analysis of the status of species that are considered alien.** A species is considered alien if introduced from another region into the north of Norway. Information sources: columns A-F (see bottom). Column A-D: alien on a national scale. Column E-F: alien on a regional scale. If left empty, no data is available from this source or species is considered native according to the definition of the source. Numbers are year of first recording, X means a species is stated as alien in the source without year of first recording. Species are used based on an alien status in at least 2 independent sources. Species in red are left out due to ambiguities in species/subspecies definition. Origin after D.
(DOCX)
