# Correction: Alien Roadside Species More Easily Invade Alpine than Lowland Plant Communities in a Subarctic Mountain Ecosystem

**DOI:** 10.1371/journal.pone.0102109

**Published:** 2014-06-30

**Authors:** The PLOS ONE Staff

There are a number of errors in the figure legends for this article. In the legends for Figures 2, 4, and 6, the grey dots incorrectly appear in black. Please view the corrected figure legends with their figures below.

**Figure 2:**
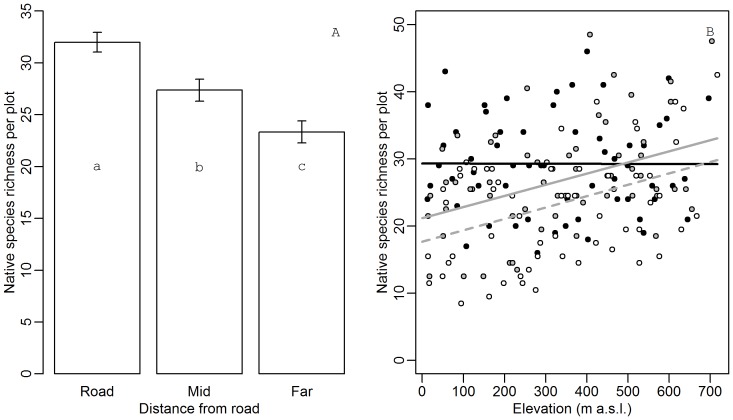
Native species richness as a function of elevation and distance to the road. (A) Average native species richness (±1 SE) in plots across the elevational gradient. Different letters indicate significant differences (p<0.05) in a Tukey’s post-hoc test. (B) Native species richness (number of species per plot) as a function of elevation. 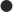, black full line: roadside plots; 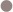, grey full line: mid plots; 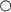, broken line: far plots (see Fig. 1 for plot types).

**Figure 4:**
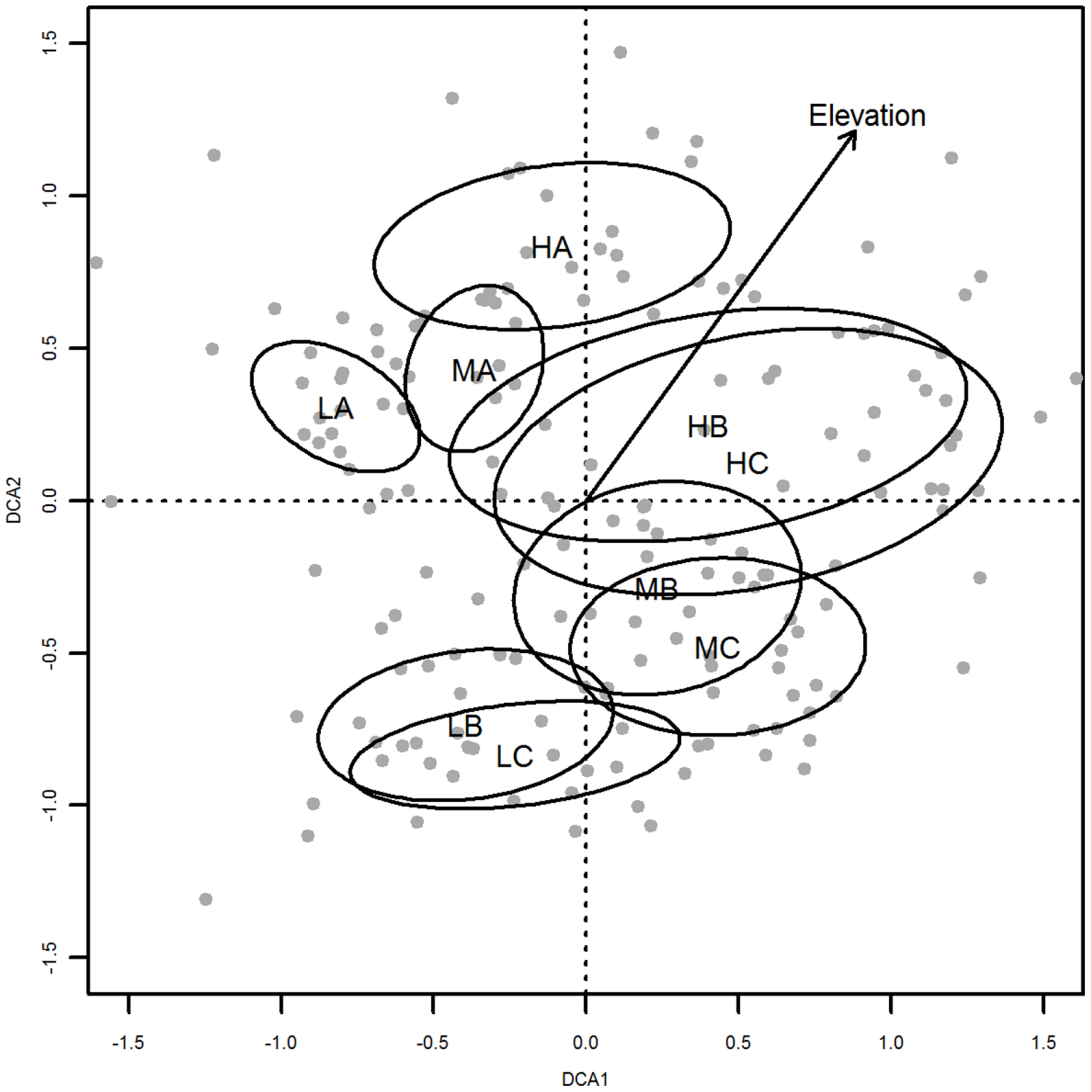
Ordination of plots. DCA-ordination of plots (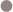) based on total species coverage. Ellipses indicate the standard deviations for different subgroups as a function of elevation and distance to the road. Elevation: H = highest third, M = middle third, L = lowest third of the gradient; road distance: A = roadside, B = mid, C = far (see Fig. 1 for plot types). The arrow represents the vector of increasing elevation. Eigenvalues of DCA1 and 2 are 0.3479 and 0.2771 respectively.

**Figure 6:**
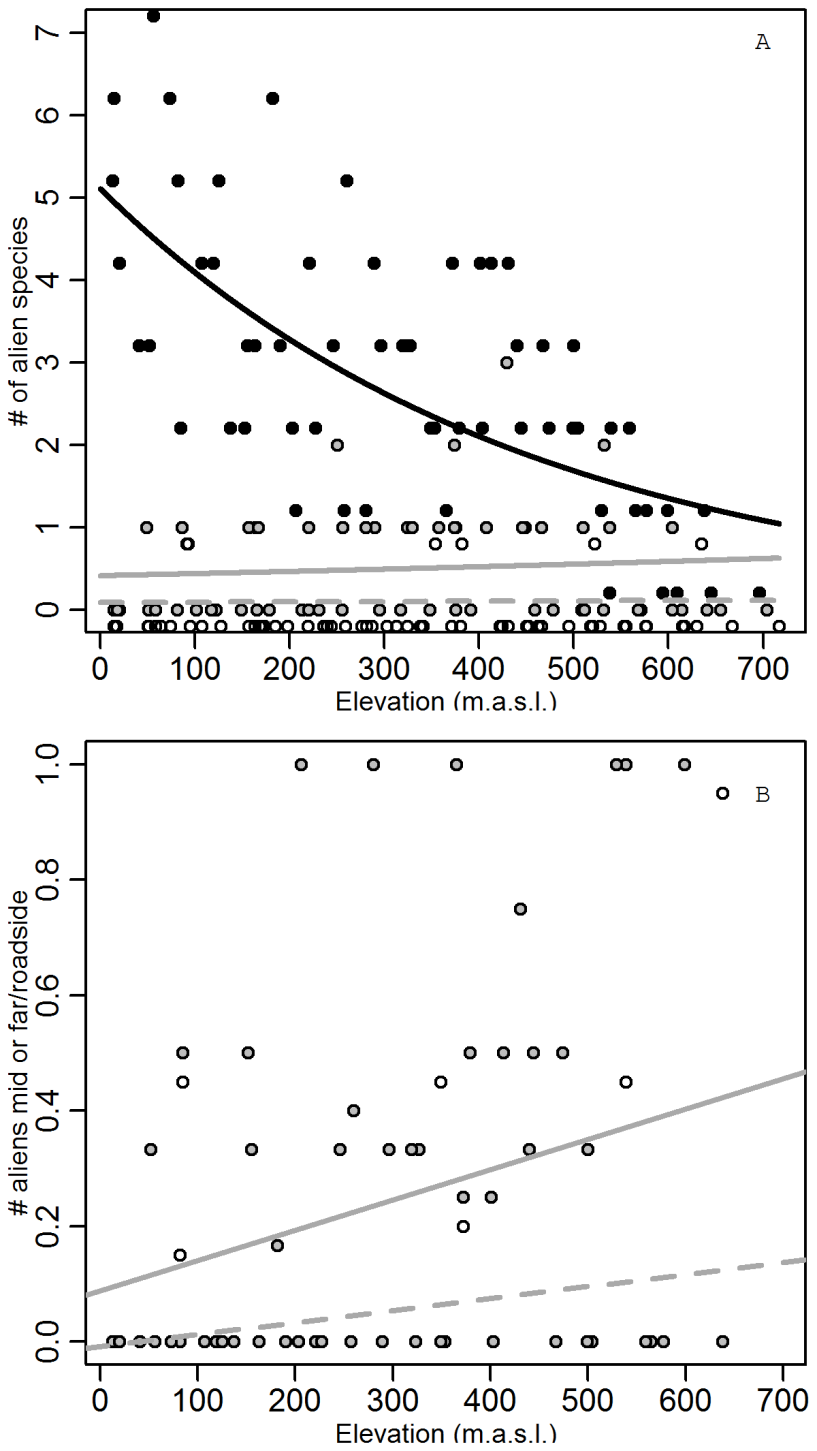
Alien species richness as a function of elevation and distance to the road. (A) Alien species richness (number of species per plot) as a function of elevation. Roadside plots (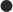, black line), intermediate plots (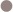, grey line) and far plots (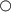, dashed grey line). (B) Ratio of alien species richness in the natural plant communities to that in the roadside plot, with mid/roadside (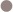, grey line) and far/roadside (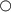, dashed grey line). Significance of linear regressions: see text. Symbols of different variables were slightly shifted to avoid overlap.
