# Supplementary material for: Alien Roadside Species More Easily Invade Alpine than Lowland Plant Communities in a Subarctic Mountain Ecosystem
Source: PLoS One. 2014 Feb 26;9(2):e89664. doi: 10.1371/journal.pone.0089664 (PMC3935920; doi:10.1371/journal.pone.0089664)
Supplement: Appendix S1 — Analysis of the status of species that are considered alien. A species is considered alien if introduced from another region into the north of Norway. Information sources: columns A-F (see bottom). Column A-D: alien on a national scale. Column E-F: alien on a regional scale. If left empty, no data is available from this source or species is considered native according to the definition of the source. Numbers are year of first recording, X means a species is stated as alien in the source without year of first recording. Species are used based on an alien status in at least 2 independent sources. Species in red are left out due to ambiguities in species/subspecies definition. Origin after D. (DOCX) [file pone.0089664.s001.docx]

**Appendix S1**

| Species names | Origin | A | B | C | D | E | F |
| --- | --- | --- | --- | --- | --- | --- | --- |
| *Achillea millefolium* | Europe |  |  |  |  | 1980 | >1600 |
| *Aegopodium podagraria* | Eurasia | <1800 | X | X | <1700 | 1900 | >1600 |
| *Agrostis capillaris* | Europe | REG |  |  |  |  | >1600 |
| *Anthoxanthum odoratum* | Europe |  |  | X |  |  | >1900 |
| *Festuca pratensis* | Europe |  |  | X | <1700 | 1950 |  |
| *Phleum pratense ssp. pratense* | Eur., Sib., | <1800 | X | X | <1700 | 1990 | >1900 |
| *Picea abies* | Europe |  |  |  |  | 1950 | >1900 |
| *Plantago major* | Eurasia |  | X |  | <1700 | 1900 | >1600 |
| *Poa annua* | Europe |  | X | X | <1700 |  | >1600 |
| *Poa pratensis ssp. pratensis* | Europe |  | X |  | <1700 |  | >1900 |
| *Stellaria graminea* | Eurasia |  |  | X | 1871 |  |  |
| *Tanacetum vulgare* | Eurasia |  | X |  |  |  | >1900 |
| *Taraxacum officinale* | Europe |  | X |  | 1990 |  |  |
| *Trifolium pratense* | Europe |  | X |  | <1700 |  | >1600 |
| *Trifolium repens* | Europe |  | X | X | <1700 |  | >1600 |
| *Vicia cracca* | Circumpolar |  |  | X | 1770 |  |  |

A –[[37](#_ENREF_37)]. Norwegian Black List 2012. Species are treated as aliens if introduced in Norway by humans after 1800. However, an additional table is provided on species introduced by humans before this date.
B –[[36](#_ENREF_36)]. Norwegian Black List 2007. Species are treated as aliens if they are introduced in Norway by humans without any delimitation in time.
C – [[38](#_ENREF_38)] DAISIE. The European dataset on alien species.
D – [[39](#_ENREF_39)] Weidema et al. 2000 shows species introduced by humans in Norway, with a date of first introduction.
E – [[40](#_ENREF_40)] GBIF. A database with georeferenced species occurrence data over a period from before 1900 to now. Species patterns can be followed over years and the first recording in the northern third of Norway was marked. If a species was not found in the region before a certain time, it indicates that it was only introduced recently as alien. If occurrence data for northern Norway were already available from before 1900, no value is displayed in the table.
F – [[41](#_ENREF_41)] Expert advice from Torbjørn Alm, University of Tromsø, distinguishing two types of aliens: those that are introduced only recently in the northern part of Norway (> 1900) and those that were introduced by humans in the lowlands earlier (>1600), but that only started invading the mountains recently.
